# An Improved Rosin Transfer Process for the Reduction of Residue Particles for Graphene

**DOI:** 10.1186/s11671-020-03312-1

**Published:** 2020-04-17

**Authors:** Kashif Shahzad, Kunpeng Jia, Chao Zhao, Xiangyu Yan, Zhang Yadong, Muhammad Usman, Jun Luo

**Affiliations:** 1 Key Laboratory of Microelectronic Devices & Integrated Technology, Institute of Microelectronics, Chinese Academy of Sciences, Chaoyang District, Beijing, 100029 People’s Republic of China; 2 School of Microelectronics, University of Chinese Academy of Sciences (UCAS), Beijing, 100049 People’s Republic of China; 3 National Center for Physics, Quaid-i-Azam University, Islamabad, 46000 Pakistan

**Keywords:** Graphene, Rosin transfer process, Sheet resistance

## Abstract

In this work, an improved rosin transfer process is initiated. An anisole coating is introduced based on the rosin transfer process to reduce the residue particles on the surface of transferred graphene. Rosin/graphene and anisole/rosin/graphene samples are handled without baking and with baking at different temperatures, i.e., 100 °C, 150 °C, and 200 °C. Atomic force microscopy (AFM) and Raman spectroscopy are employed to characterize the surface properties of transferred graphene. The removal of the protective rosin layer and anisole/rosin layers without baking is found to be more effective and beneficial compared to the conventional PMMA transfer process. Furthermore, better results in terms of reduced surface roughness and residue particles are accomplished by introducing anisole in the improved rosin transfer process. Uniform and low sheet resistance (*R*_sh_) is also observed across transferred graphene using this improved process.

## Background

The isolated two-dimensional (2D) nature of graphene has attracted tremendous interest due to its exceptional properties. However, these excellent properties are attributed to the isolated single-layer graphene. Such unique properties include mechanical breaking strength of ~ 130 GPa [1] and unusual electrical properties [2–4] compared to other semiconductor materials, i.e., electron mobility beyond 2.5 × 10^5^ cm^2^ V^−1^ s^−1^ at room temperature [5]*.* Based on aforementioned rare properties, graphene has become one of the most promising alternatives for Si. All of these features make graphene to step into the new generation of technologies beyond the limitations of conventional semiconductor materials [6–8].

The properties described above are mostly related to intrinsic graphene. In reality, to achieve these complex properties, large area growth of graphene is required. For the growth of graphene, chemical vapor deposition (CVD) method is an efficient and inexpensive process for producing large area single-layer graphene [9]. However, it requires a metal substrate such as Cu using the CVD method to grow graphene. The full use of excellent properties of graphene requires as-grown graphene to be transferred onto a variety of substrates. Since CVD-grown graphene is more attractive for the application in high-performance electronic devices and transparent electrodes [10, 11], different methods, therefore, have been developed to transfer it onto the insulating material, such as polydimethylsiloxane (PDMS) [12], polymethyl methacrylate (PMMA) [13–16], and polycarbonate (PC) [17]. and followed by the removal of these polymers through dissolution in organic solvents. Nevertheless, despite intensive care has been paid into such methods, the strong interaction between polymers and graphene as well as the low solubility of polymers in solvents, unfortunately, makes it pretty difficult to remove polymer residues completely. The remaining polymer residues and damage for as-transferred graphene inevitably degrade the performance of graphene-based devices significantly. Therefore, the resulting surface roughness and damage of as-transferred graphene impose a major challenge in improving the optical, electrical, and mechanical properties of graphene [18, 19]. In order to make full use of these properties, a scalable transfer method in which the requirements of fewer impairments and polymer free can be satisfied is highly desired.

To meet these requirements, the first need is to study the reason for impairments on the surface of graphene. The impairments mainly result from the removal of the protective polymer layer in solvents. The purpose of this polymer protective layer is to protect the graphene from folding, tearing, and cracks. A good protective layer should have low adsorption energy (*E*_ad*.*_), good support strength, and good solubility in solvents and the last guarantees the easy removal of this protective layer after graphene transfer. Recently, rosin (C_19_H_29_COOH), a small natural organic molecule, was reported to provide a good protective layer with low *E*_ad_ (1.04 eV) compared to popularly used PMMA (*E*_ad_ > 1.45 eV), a good supporting strength, and, more importantly, an easy removal in solvents due to the intrinsic property as a small molecule [20]. Therefore, rosin promotes our interest to assist in clean and damage-free transfer of CVD-grown graphene immensely.

Hereby, we describe the rosin transfer of CVD-grown graphene, which is proved to be well soluble in organic solvents and has weak interaction with graphene and provides sufficient mechanical supporting strength. The glass transition temperature of rosin is 70 °C. Since appreciable polymer residues still exist using the rosin transfer process in our work, an improved rosin transfer process, in which an anisole recoating is introduced in order to reduce the polymer residues remarkably, is proposed. Moreover, before dipping into acetone to dissolve the protective polymer layer on graphene, i.e., rosin and anisole/rosin, samples are baked at 100 °C, 150 °C, and 200 °C for 30 min in order to probe if baking has effects on removing polymer residues and improving surface roughness of as-transferred graphene. The results were compared with prevailing PMMA transfer process.

## Presentation of the Hypothesis

**Fig. 1.**
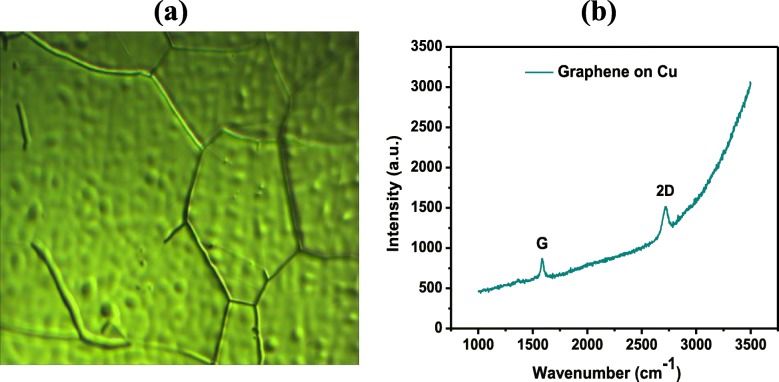
**a** Optical micrograph of CVD-grown graphene on Cu. **b** Raman spectroscopy of CVD-grown graphene on Cu

The graphene samples employed here were grown on a 25-μm-thick copper (Cu) foil (5 × 5 cm^2^) by low pressure chemical vapor deposition (LPCVD) in a quartz tube furnace [21, 22]. Initially, the copper foil was annealed in hydrogen atmosphere at 1010 °C and 300 Pascal pressure for 1 h. Then, the decomposition of precursor (CH_4_:H_2_ = 0.5:300 sccm) was flowed in the furnace at the same temperature/pressure for 50 min to grow thin crystalline film of graphene. After the synthesis, graphene samples were cooled down to room temperature (the flow of methane was stopped at 600 °C). However, the carbon dissolves in metal up to a few atomic percent; the use of non-carbide forming metals, e.g., Cu, Ni, and Pt, is preferred [23]. The commonly used metals are Ni and Cu, which both act as a catalyst. Although Ni is cheaper than Cu, it is found that the thermal catalytic decomposition of methane on copper foil is a self-limiting process. In this case, it has been reported that 95% of the substrate surface is covered by graphene [21]. Therefore, Cu becomes the popular selection as the substrate material for CVD-grown single-layer graphene. Figure 1 shows the optical microscope image and Raman spectra of CVD-grown graphene.

**Fig. 2:**
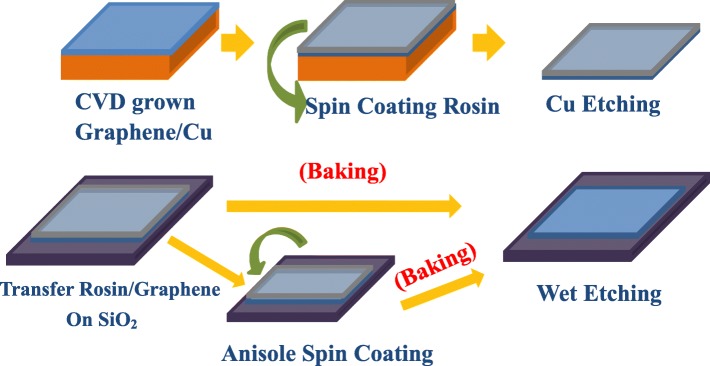
Schematics of transfer process

Figure 2 illustrates the schematics of rosin transfer and improved rosin transfer processes, respectively. Rosin was spin-coated on the CVD-grown graphene as a shield to protect from damage during the transfer process. The 50 wt. % solution of rosin (C_19_H_29_COOH) dissolved in ethyl lactate was used because of high viscosity and good film forming ability. Note that the employment of rosin with concentration less than 50 wt. % usually leads to less viscous, smother, and low film forming ability which cannot offer sufficient support for graphene. The rosin/graphene/Cu samples were then placed in cleansing solution (HCl:H_2_O_2_:H_2_O = 1:1:1) for 50 s to remove the dust and the residues attached on the back side of Cu during the spin coating. The accessible graphene-copper face was then etched by immersing in a marble solution HCl (50 ml):H_2_O (50 ml):CuSO_4_·5H_2_O (10 g) for 1.5 h, leaving behind a pliant membrane of rosin/graphene suspended in the solution. The suspended membrane was transferred to DI water for 5 times to rinse residual etching solution. The floating flexible and fragile membrane was transferred on the SiO_2_ substrate with care and precision. A modified rosin transfer process was proposed to further reduce the polymer residues and to improve the quality of transferred graphene, where rosin/graphene/SiO_2_ samples were spin-coated with anisole at 500 rpm for 10 s and at 1200 rpm for 30 s. All samples were categorized into not baked (room temperature, RT) and baked at 100 °C, 150 °C, and 200 °C for 30 min. The supporting rosin layer is removed by acetone bath, while anisole is used in the improved rosin-enabled transfer process which was also then removed by acetone bath. All transferred graphene was characterized using the Raman spectroscopy at 532 nm excitation wavelength in air using × 100 objective to determine the quality of pristine and as-transferred graphene layer using the improved rosin-enabled transfer process. The AFM characterization is done in tapping mode using the Bruker Dimension Icon model at standard temperature and atmosphere conditions. The four-point measurement (Kelvin technique) is performed to measure the sheet resistance at random points on the 2 × 2 cm^2^ area of samples.

## Testing the Hypothesis

**Fig. 3:**
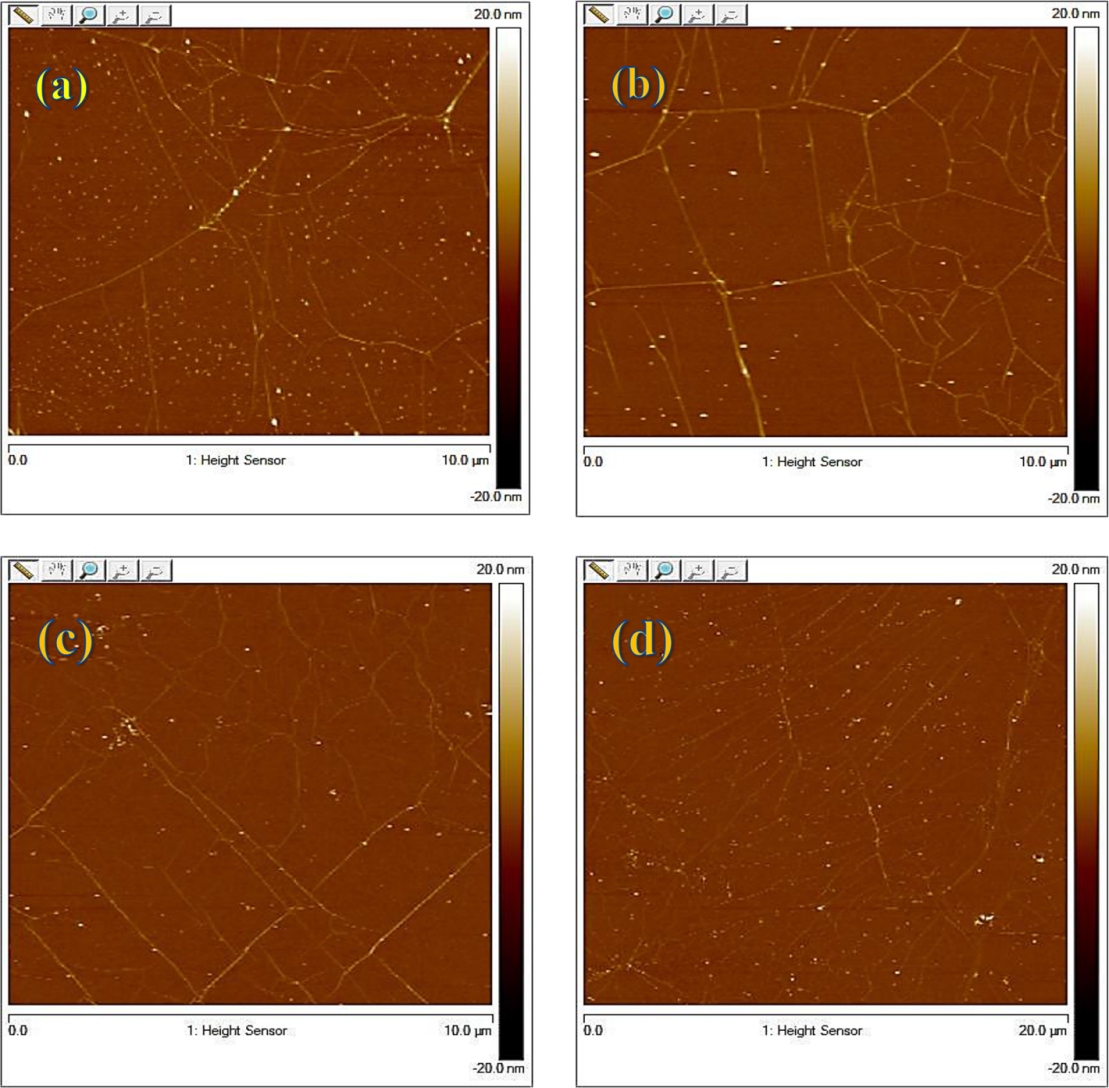
AFM spectrum of **a** rosin/graphene coated transfer at room temperature (RMS = 0.668 nm) and **b**–**d** rosin/graphene sample baked at 100 °C (RMS = 0.889 nm), 150 °C (RMS = 0.97 nm), and 200 °C (RMS = 0.992 nm), respectively

Figure 3 shows the AFM images of graphene using the rosin transfer process without baking described here as at room temperature (RT) and with baking at different temperatures, i.e., 100 °C, 150 °C, and 200 °C for 30 min, respectively. The surface morphology of as-transferred graphene was investigated using AFM in close contact (tapping) mode and standard atmospheric conditions. As seen, there are visible wrinkles on the surface of all graphene samples which cannot be avoided as long as CVD-grown graphene on Cu is utilized. Apart from wrinkles, some rosin residues tend to remain on the surface, which are visible as white dots in the AFM spectrograph image. If scrutinized, the RT case shows the most particles in contrast to others with baking. This demonstrates clearly that the baking is useful in reducing residue particles in the rosin transfer process. The root mean square (RMS) and roughness (*R*_q_) values of as-transferred graphene are also collected by scanning surface area of 10 μm × 10 μm. Compared to *R*_q_ values of 0.889 nm, 0.97 nm, and 0.992 nm for graphene baked at 100, 150, and 200 °C, the lowest *R*_q_ value of 0.668 nm occurs for the graphene without baking. This, however, points out that baking is not beneficial in achieving a low *R*_q_ value which is also desired for practical device application of graphene. This *R*_q_ value can be especially used as the quantification of surface morphology of transferred graphene. The water molecules trapped between pliant graphene membrane and SiO_2_ during pickup from DI water would rupture the graphene, hence producing cracks within the graphene. As a result, the *R*_q_ value increases with increasing baking temperature. It is, therefore, not recommended to bake graphene at high temperatures even if baking is good at reducing residue particles.

**Fig. 4:**
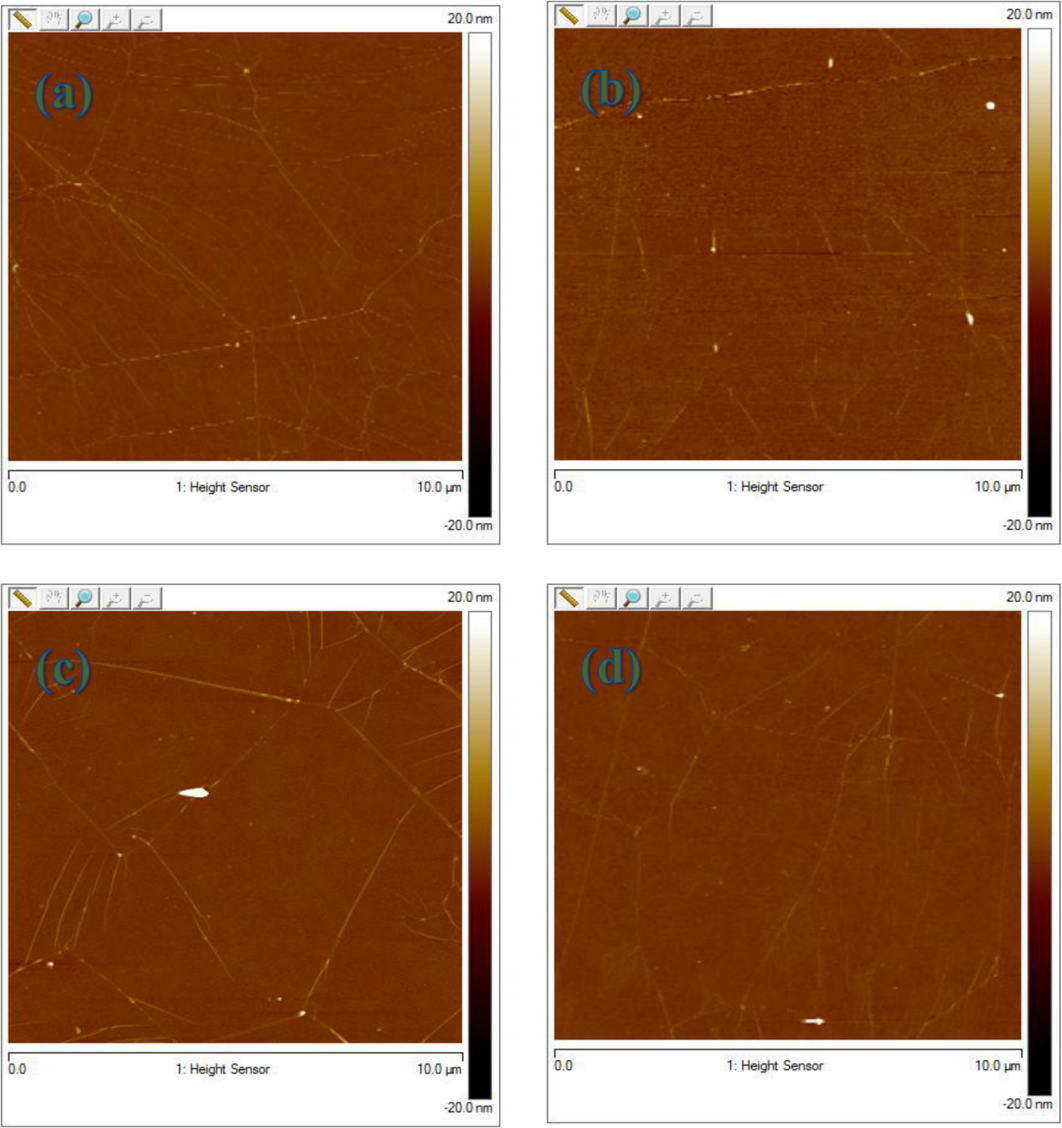
AFM spectrum of **a** anisole/rosin/graphene coated transfer at room temperature (RMS = 0.523 nm) and **b**–**d** anisole/rosin/graphene sample baked at 100 °C (RMS = 0.887 nm), 150 °C (RMS = 0.950 nm), and 200 °C (RMS = 0.98 nm), respectively

**Fig. 5:**
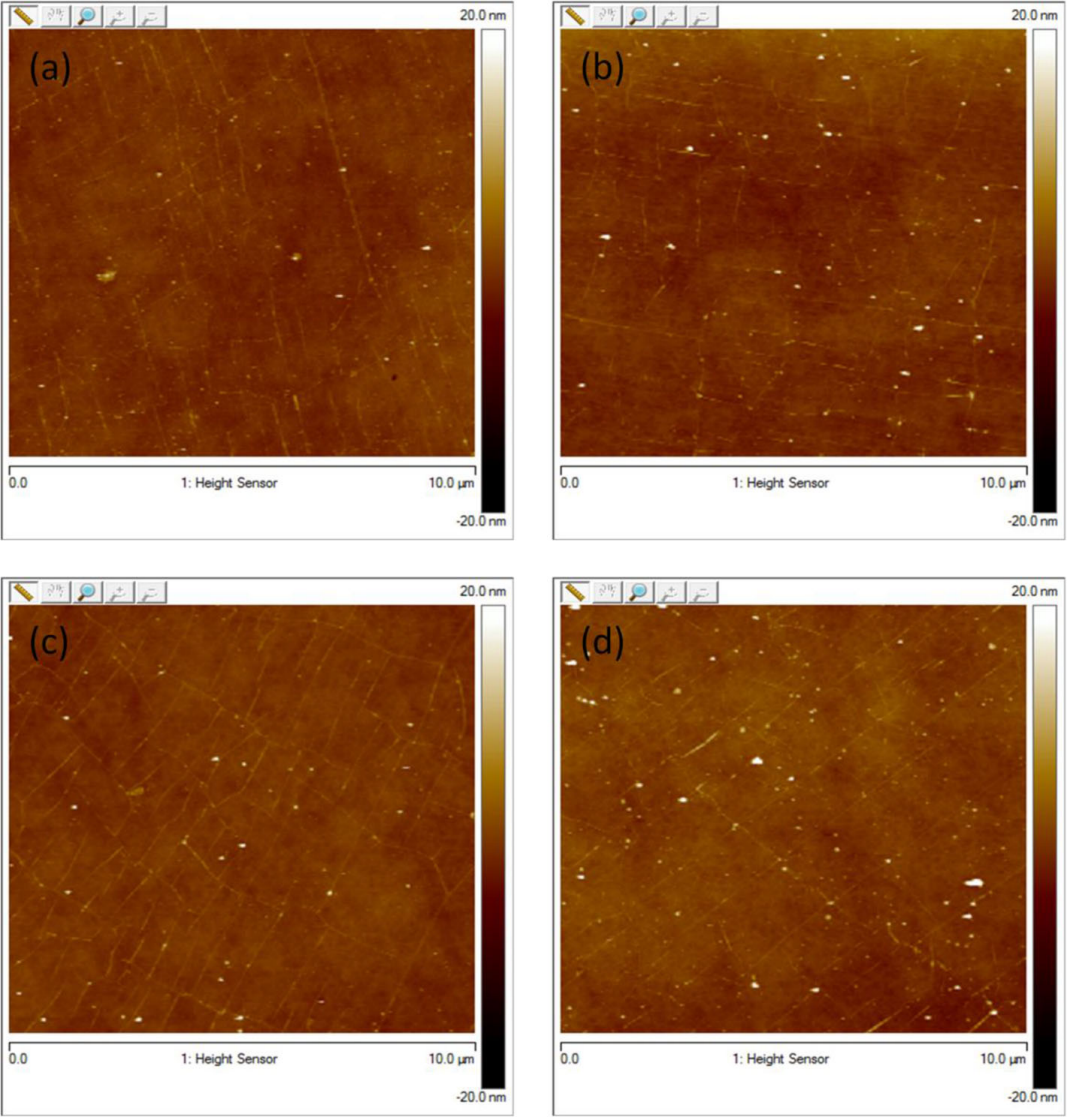
AFM spectrum of **a** PMMA coated transfer at room temperature (RMS = 1.03 nm) and **b**–**d** PMMA transferred sample baked at 100 °C (RMS = 1.51 nm), 150 °C (RMS = 1.49 nm), and 200 °C (RMS = 1.72 nm), respectively

**Fig. 6:**
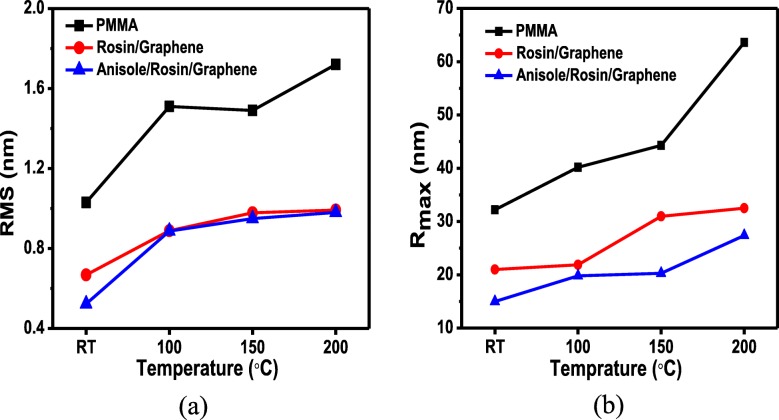
**a** Root mean square (RMS) (nm) roughness of PMMA, rosin/graphene, and anisole/rosin/graphene. **b** Maximum height (*R*_max_) of PMMA, rosin/graphene, and anisole/rosin/graphene coated transfer

Figure 4 shows the AFM images of graphene using the improved rosin transfer process in the presence of anisole without baking (RT) and with baking at different temperatures, i.e., 100 °C, 150 °C, and 200 °C for 30 min, respectively. As seen, wrinkles are also observed for all transferred graphene but the visibility is weaker compared to only rosin-enabled transfer process in Fig. 3 and PMMA-enabled transfer process in Fig. 5. As anticipated, the residue particles are greatly decreased for all graphene in sharp contrast to the observations in Fig. 3. In the improved rosin transfer process, this remarkable reduction of residue particles with the introduction of anisole would rather be attributed to the capability of anisole as a strong solvent in collaboration with acetone. Anisole/rosin dissolves more easily than bare rosin in acetone which leads to cleaner graphene in the improved rosin transfer process. In addition, the *R*_q_ values for graphene without baking and with baking at 100, 150, and 200 °C are 0.523 nm, 0.887 nm, 0.95 nm, and 0.98 nm, respectively. A relaxation to as-transferred graphene with the introduction of anisole may help in achieving the lower *R*_q_ value of 0.523 nm in the improved rosin transfer process than that of 0.668 nm in the rosin transfer process, while the lowest value for *R*_q_ in case of conventional transfer method using PMMA is 1.03 nm. In this improved rosin transfer process, it is again proved that the baking is not beneficial in achieving a low *R*_q_ value because of similar reason, i.e., cracks produced during the baking at high temperature. Note that compared to the *R*_q_ value of 1.03 nm in the PMMA transfer process, both the rosin and improved rosin transfer process show much smaller *R*_q_ values, which manifests the superiority of adopted graphene transfer processes in this work. Compared with *R*_q_ roughness, the maximum height of large residual particles (*R*_max_) is also an important parameter in the application of large area thin film devices, because it determines whether short circuit may occur in devices. Figure 6b shows the average *R*_max_ at room temperature, 100 °C, 150 °C, and 200 °C. The minimum value for the *R*_max_, i.e., 15 nm, is achieved at RT for anisole/rosin/graphene. This also confirms the advantage of improved rosin transfer process at RT.

**Fig. 7:**
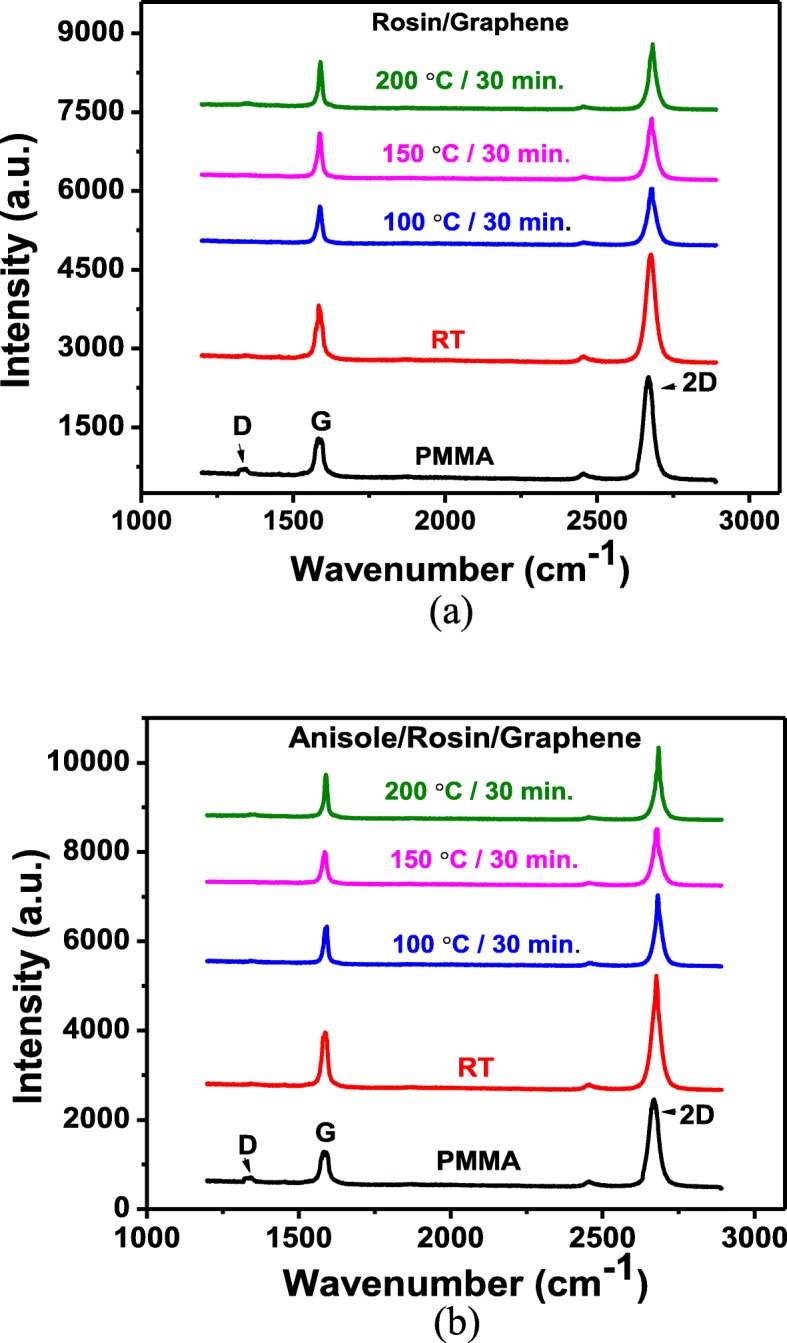
**b** Raman spectrum of rosin/graphene coated transfer at different temperatures compared to PMMA transfer. **b** Raman spectrum of anisole/rosin/graphene coated transfer at different temperatures compared with PMMA transfer

Despite that the improved rosin transfer process is obviously advantageous in terms of residue particles and *R*_q_ values and *R*_max_, the quality of as-transferred graphene deserves to be evaluated. In Fig. 7, the Raman spectra of as-transferred graphene using the rosin and improved rosin transfer process without baking (RT) and with baking at 100 °C, 150 °C, and 200 °C are displayed. As seen in Fig. 7a, two peaks situated in the Raman spectra at 1580 cm^−1^ (G), a primary in-plane vibrational mode, and 2676 cm^−1^, a second-order overtone of a different in-plane vibration (2D), are found. These peaks are adduced from a 532-nm excitation laser. The position and shape of these two peaks are prominent, clearly defining the material to be graphene. Also, the ratios of 2D band to G band intensities (*I*_2D_/*I*_G_) are 1.61 to 1.65, indicating the single layer of as-transferred graphene. The absence of D peaks in the Raman spectra for as-transferred graphene with baking at different temperatures confirms that the disorder is unlikely to appear using both the rosin and improved rosin transfer process. Also, no rosin- and anisole-related peaks are detected for all transferred graphene. The assumption no rosin- or anisole-related peaks was made on the fact that the Raman spectra appeared to be same after transfer process as those observed compared to the Raman spectra of pristine graphene grown on Cu. The appearance of D peak after transfer process in the baked sample shows the induced defects during the removal of rosin. Furthermore, the rosin residues after transfer process are very low. Therefore, rosin-related peaks are unlikely to appear in the Raman spectra of as-transferred graphene.

Shifts in both the G and 2D Raman peaks of graphene are usually produced by a combination of strain and doping due to the interaction with the substrate or support layer during transfer process. It is known that the blue shift of both the G band and 2D band positions indicated p-doping of graphene. The entailed 2D peak upshift of ~ 6 cm^−1^ demonstrates the doping of rosin-enabled transfer process; the described phenomenon has been reported previously in the literature [24, 25]. The peak intensity for as-transferred graphene without baking is obviously higher than that with baking at high temperatures. Besides, the full width at half maximum (FWHM) value of 2D band for as-transferred graphene without baking is 38.18 cm^−1^ which is the smallest compared to those with baking at high temperatures. These results mean that room temperature is favorable for achieving high-quality graphene during the rosin transfer process.

In Fig. 7b, the Raman spectra for as-transferred graphene using the improved rosin transfer process are shown; similar observations can be made for as-transferred graphene using the rosin transfer process. The peak intensity is also very high, and the FWHM value of 2D band for as-transferred graphene without baking is 35.79 cm^−1^ which is a little bit lower than that in Fig. 7a. All aforementioned results manifest that the quality of as-transferred graphene is intact or even better using this improved rosin transfer process, compared to the rosin transfer process.

**Fig. 8:**
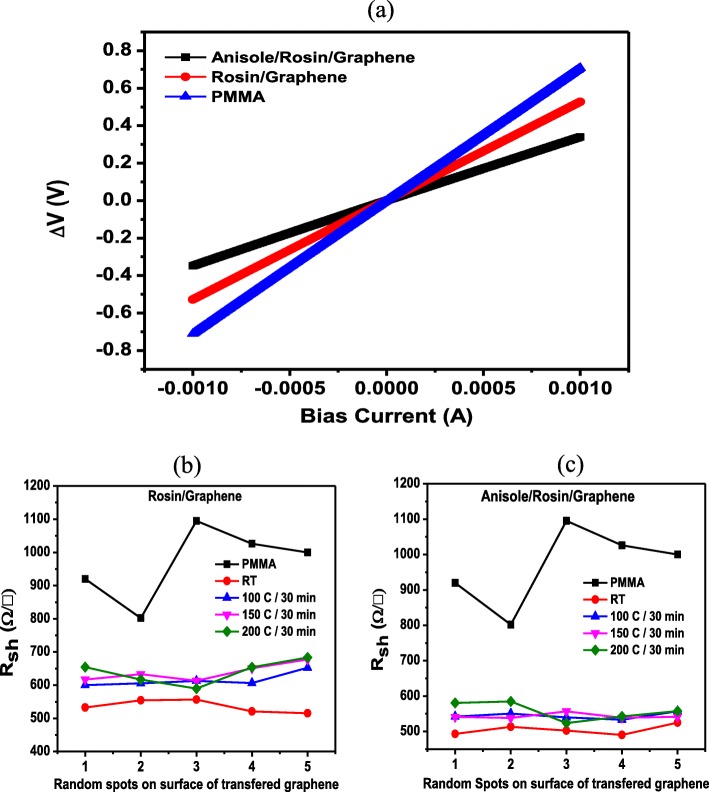
**a** I–V characteristic curve for typical transferred graphene by using PMMA, rosin, and anisole/rosin graphene. **b** Sheet resistance *R*_sh_ measurement at 5 different random spots of as-transferred graphene by rosin/graphene. **c** Sheet resistance measurement at 5 different random spots of as-transferred graphene by anisole/rosin/graphene

**Fig. 9:**
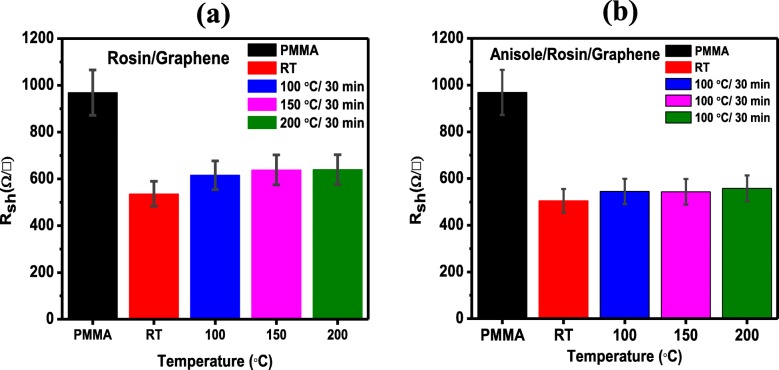
**a** Average value of the sheet resistance measurement of rosin-enabled transfer process. **b** Average values of the sheet resistance of improved rosin-enabled transfer process with the minimum sheet resistance value of 493.4 Ω/□ at RT

Figure 8a, illustrates the I–V characteristics of the as-transferred graphene using the PMMA, rosin, and anisole/rosin transfer process. To double check the quality of as-transferred graphene, the sheet resistance (*R*_sh_) data are collected and illustrated in Fig. 8b, c. The sheet resistance was measured by a 4-probe resistivity measurement system. Moreover, this is an essential and main metric of electrical performance for 2D materials. *R*_sh_ is measured at 5 points on each sample. The size of the sample is about 2 × 2 cm^2^ in order to get reliable results. In Fig. 8b, the *R*_sh_ data for as-transferred graphene using the rosin transfer process at random spots are presented. As seen, for all graphene, scattered *R*_sh_ values in the range of 500–700 Ω/□ are found across the surface of as-transferred graphene. The lowest value of *R*_sh_ occurs for the graphene without baking which is also in good agreement with the observations from the Raman spectra. In Fig. 8c, the *R*_sh_ values for as-transferred graphene using the improved rosin transfer process are shown. As seen, compared to Fig. 8a, the uniformity of *R*_sh_ is much better and the range of *R*_sh_ values is significantly narrower, i.e., 500–600 Ω/□. More importantly, the *R*_sh_ values in the improved rosin transfer process are generally lower than those in the rosin transfer process and the lowest *R*_sh_ value of ~ 500 Ω/□ also happens for the graphene without baking. Figure 9a, b shows the average value of sheet resistance across the sample surface. The bar chart clearly shows the average value of sheet resistance for the improved rosin transfer process is the lowest, i.e., 493.4 Ω/□. This demonstrates again the superiority of this improved transfer process proposed in the present work in terms of electrical performance. Of course, it is worth noting that apart from improved electrical performance, the changes in sheet resistance could be also a result of other factors such as doping.

## Implications of the Hypothesis

In this work, an improved rosin transfer process is proposed for the purpose to reduce residue particles further on the basis of the rosin transfer process. The established improved transfer process is compared with the conventional PMMA transfer process. It is found that this improved rosin transfer process by the introduction of anisole is indeed advantageous in terms of significantly reduced residue particles as well as good quality of transferred graphene. This remarkable reduction of residue particles would rather be attributed to the capability of anisole as a strong solvent in collaboration with acetone. Anisole/rosin dissolves more easily than bare rosin in acetone which leads to cleaner graphene in this improved rosin transfer process. The FWHM value of 2D band for as-transferred graphene using the improved rosin transfer process is 35.79 cm^−1^, which is obviously lower than 38.18 cm^−1^ for transfer graphene using the rosin transfer process. In addition, as-transferred graphene using the improved rosin transfer process shows generally lower *R*_sh_ values of 500–600 Ω/□ than those of 500–700 Ω/□ using the rosin transfer process. The baking at high temperatures is found to exert marginal effects on residue particles and quality for as-transferred graphene which is thus not recommended. Achieved results in this work are ought to be helpful in advancing clean graphene transfer process in order to realize graphene-based devices of high performance in the future.

## Data Availability

Authors declare that the materials, data, and associated protocols are available to the readers, and all the data used for the analysis are included in this article.
